# Surgical treatment of left ventricular fibroma accompanied with ventricular septal defect in an infant: a case report

**DOI:** 10.1186/1749-8090-9-37

**Published:** 2014-02-20

**Authors:** Yibo Gong, Ni Yin, Jinfu Yang

**Affiliations:** 1 Department of the cardiothoracic surgery of the 2nd Xiangya Hosptial, Central South University, 410011 Changsha, China

**Keywords:** Congenital heart disease, Infant, Cardiac fibroma

## Abstract

Primary cardiac tumor in infancy is an uncommon condition which is rarely accompanied with congenital heart disease. Although these tumors are generally benign, the complication associated with it, such as arrhythmia, outflow tract obstruction, and heart failure, may result in early mortality, when combined with congenital heart disease. Early surgical treatment may reduce complication risk and increase operative success rate which may improve the patient’s long-term prognosis. Herein, we report a case of an eight-month-old Chinese baby boy diagnosed with a calcified fibroma combined with ventricular septal defect, the tumor excision and repair of ventricular septal defect were done at the same time.

## Background

Primary cardiac tumors in infancy and childhood are uncommon, and approximately 70% of them are benign with morbidity rate of around 0.27% to 0.8%. Most specialists advocate early individualized surgical resection to reduce mortality caused by complications such as heart inflow/outflow tract obstruction, and malignant arrhythmia. Up to now, cardiac fibroma accompanied with VSD and PH has never been reported before, let alone simultaneous surgical treatment of both conditions.

## Case presentation

**Figure 1:**
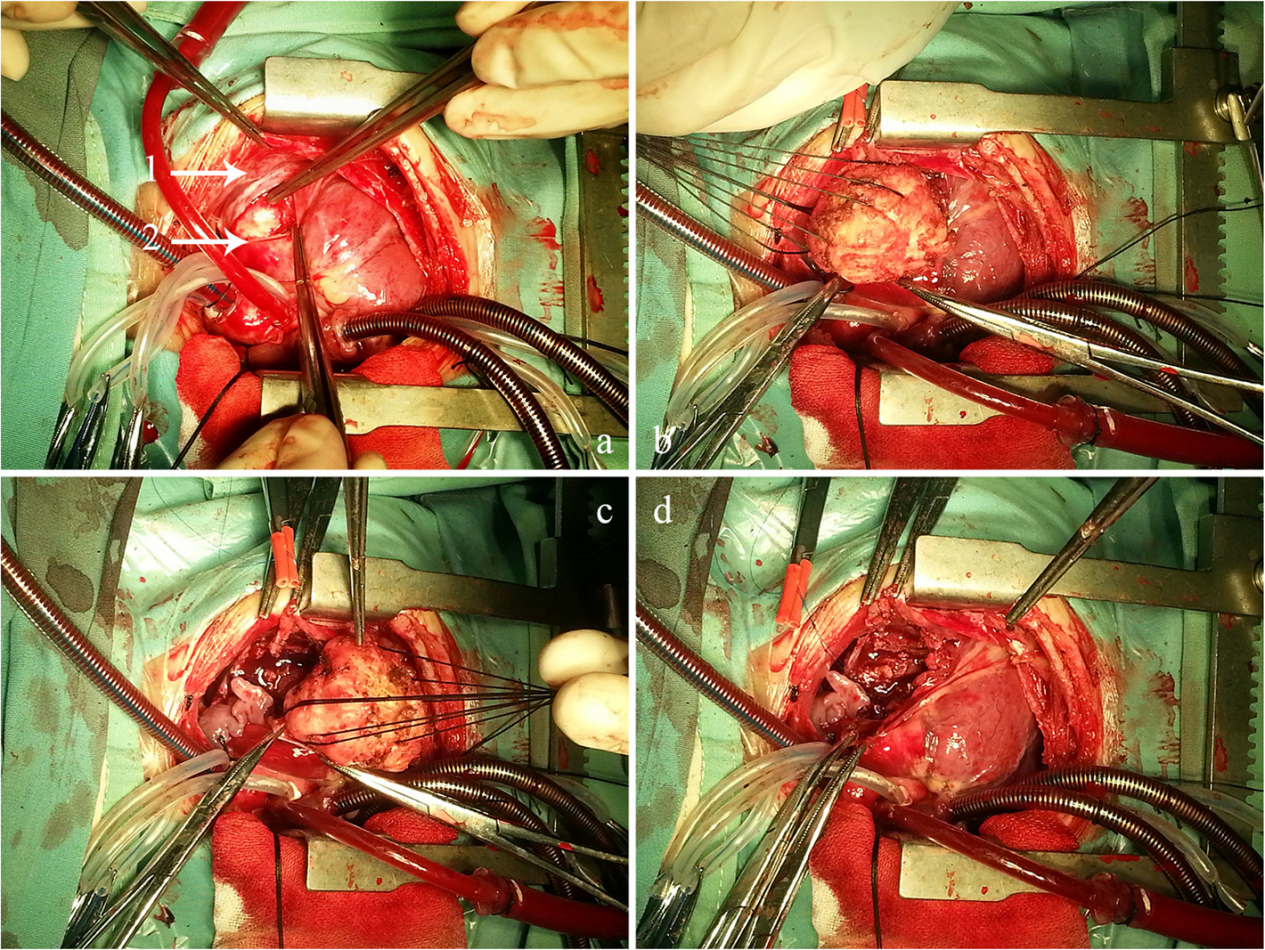
**Surgical exploration reveals a firm 4 cm × 3.5 cm × 3 cm mass with unclear-edge under the LCX (1) and LAD (2).** The cardiac mass, which was supplied by a large branch from LCX was surgically removed. 4 panels **(a,b,c,d)** show the whole process how the mass removed from LV.

An eight-month-old Chinese baby boy was admitted with a heart murmur suggestive of VSD. Echocardiogram revealed VSD and pulmonary hypertension (PH), the tumor was not detected until the operation. The patient subsequently underwent surgery under cardiopulmonary bypass (CPB). During the operation, the patient’s pulmonary artery was noticeably dilated, and a perimembranous VSD approximately 16 mm in diameter was revealed. Further surgical exploration discovered a firm 4 cm × 3.5 cm × 3 cm mass with unclear-edges under the junction of the left anterior descending (LAD) artery and the left circumflex (LCX) artery (Figure 1a). The mass was supplied by a large branch originating from LCX, and was adherent to surrounding tissues. Despite the mass being asymptomatic, there were concerns for the continued growth causing coronary artery compression and left ventricular cavity obstruction. These concerns were the reason for tumor resection at the time of VSD closure.

**Figure 2:**
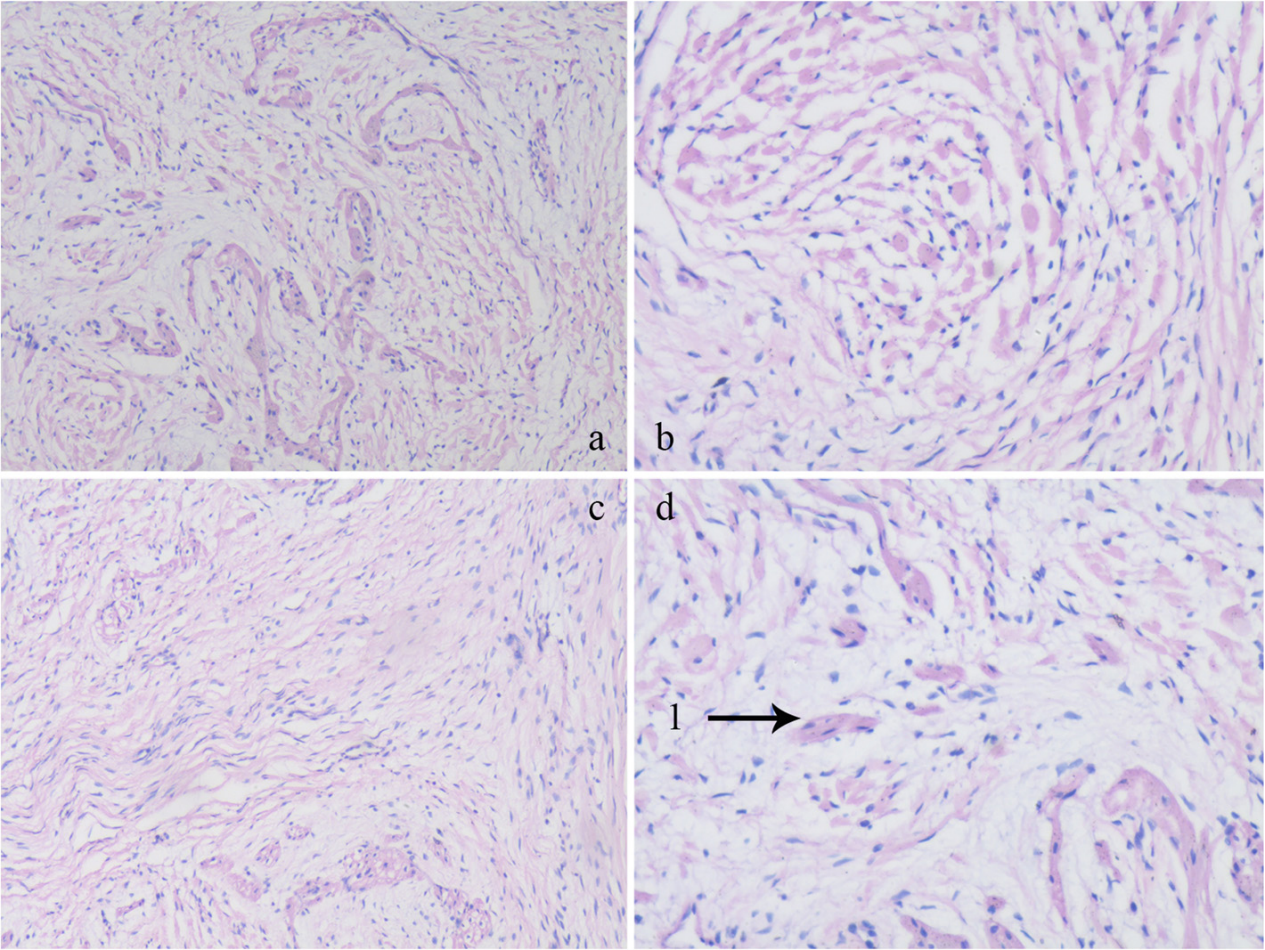
**Pathological examinations of the removed mass report revealing calcifying fibroma (Armed by the arrow).** 4 panels **(a,b,c,d)** show tumor tissue under microscope in different multiples and different position. The graphs which show normal tissue around the tumor also prompts the tumor has been completely resected.

The VSD was repaired with a teflon patch. We opened the epicardium between LAD and LCX to expose the tumor and separate it from cardiac tissues, staying well away from the coronary arteries. We finally completely excised it from the left ventricle successfully (Figure 1b, c, d). Deciding on how to reconstruct the left ventricle was then considered. A patch repair may result in ventricular aneurysm which can lead to dyssynchronous contractility and subsequent decrease in cardiac function. The left ventricle was enlarged due to the volume overload on the left ventricle caused from the left to right shunt created by the VSD. As a result the myocardial tissue could be reapproximated easily to close the left ventricular wall defect. The patient was separated from CPB without any incidents. There was good left ventricular function with no compromise of the LAD and LCX (Figure 2). The patient was discharged on post-operative day 9 and remains asymptomatic on last follow-up.

## Discussion

Primary cardiac tumors in infancy and childhood are uncommon, and approximately 70% of them are benign with morbidity rate of around 0.27% to 0.8% [1]. Cardiac fibromas, which normally arise from heart fibroblasts or myofibroblast, accounts for around 2% to 5% of all benign tumors in the heart [2]. Because of the complications such as heart inflow/outflow tract obstruction, and malignant arrhythmia associated prognosis of cardiac fibromas, many specialists advocate early individualized surgical resection [3-5]. Up to now, cardiac fibroma accompanied with VSD and PH has never been reported before, let alone simultaneous surgical treatment of both conditions. Left ventricular tumor excision combined with heart malformation correction is more complex than simple tumor excision because of prolonged CPB time, greater operation trauma.

## Conclusion

Early tumor resection is recommended in such case to guarantee a successful surgical outcome with less potential complications. Secondly our experience showed that a combined surgical procedure to excise a cardiac tumor and repair a congenital heart defect is feasible and safe.

## Consent

Written informed consent was obtained from the patient’s parents for publication of this Case report and any accompanying images. A copy of the written consent is available for review by the Editor-in-Chief of this journal.

## Abbreviations

CHD: Congenital heart disease; VSD: Ventricular septal defect; CPB: Cardiopulmonary bypass; LAD: Left anterior descending; LCX: The left circumflex; PH: Pulmonary hypertension.

## Competing interests

The authors declare that they have no competing interests.

## Authors’ contributions

GYB participated in operation as an assistant and wrote the manuscript. NY managed the patient during operation period as attending doctor. JFY performed the surgery and supervised manuscript redaction. All authors read and approved the final manuscript.

## References

[B1] Allen HD Driscoll DJ Shaddy RE Feltes TF FR DS Moss and Adams' heart disease in infants, children, and adolescents: Including the fetus and young adult 2007 7 Philadelphia: Lippincott Williams & Wilkins

[B2] Burke A Jeudy J Jr Virmani R Cardiac tumours: an update Heart 2008 9 1 117 123 10.1136/hrt.2005.078576 18083956

[B3] Horovitz A van Geldorp IE Roubertie F Thambo J-B Large right ventricular fibroma in a 6-month-old infant Pediatr Cardiol 2012 1 3 10.1007/s00246-012-0390-9 PMC3508277 22644419

[B4] Elahi M Poh C-L Krishna A Grant P Surgical considerations for large asymptomatic cardiac fibromas in the context of fatal ventricular arrhythmias Heart, Lung and Circulation 2012 9 11 750 753 10.1016/j.hlc.2012.03.005 22503173

[B5] Torimitsu S Nemoto T Wakayama M Okubo Y Yokose T Kitahara K Literature survey on epidemiology and pathology of cardiac fibroma Eur J Med Res 2012 9 1 5 10.1186/2047-783X-17-5 22472419 PMC3351722

